# Mitophagy in Diabetic Kidney Disease

**DOI:** 10.3389/fcell.2021.778011

**Published:** 2021-12-10

**Authors:** Xiaofeng Zhang, Jing Feng, Xia Li, Dan Wu, Qian Wang, Shuyu Li, Changhua Shi

**Affiliations:** School of Basic Medical Sciences, Beijing University of Chinese Medicine, Beijing, China

**Keywords:** diabetic kidney disease, mitophagy, PINK1, parkin, mechanisms

## Abstract

Diabetic kidney disease (DKD) is the most common cause of end-stage kidney disease worldwide and is the main microvascular complication of diabetes. The increasing prevalence of diabetes has increased the need for effective treatment of DKD and identification of new therapeutic targets for better clinical management. Mitophagy is a highly conserved process that selectively removes damaged or unnecessary mitochondria *via* the autophagic machinery. Given the important role of mitophagy in the increased risk of DKD, especially with the recent surge in COVID-19-associated diabetic complications, in this review, we provide compelling evidence for maintaining homeostasis in the glomeruli and tubules and its underlying mechanisms, and offer new insights into potential therapeutic approaches for treatment of DKD.

## Introduction

Diabetic kidney disease (DKD), the main microvascular complication of diabetes, is most often the consequence of chronic kidney disease and end-stage renal disease. Clinically, the main characteristics of DKD are albuminuria and decline in glomerular filtration rate (Tuttle et al., 2014). Metabolic and hemodynamic changes in diabetes lead to alterations in the glomerular structure, such as progressive thickening of the glomerular basement membrane (GBM), expansion of the extracellular matrix (ECM), loss of Sertoli cells, and typical glomerular sclerosis (Kanwar et al., 2011). Although pathogenic mechanisms of DKD remain unclear, several factors are considered to be involved in the pathogenesis of diabetic nephropathy including the generation of advanced glycation end products (Saxena et al., 2019), reactive oxygen species (ROS) (Lindblom et al., 2015; Han et al., 2018), endoplasmic reticulum stress (Fan et al., 2017; Umanath and Lewis, 2018), and inflammatory factors and activation of the renin-angiotensin system. An increasing number of studies have shown that impaired mitochondrial function, reduced mitochondrial DNA, accumulation of damaged mitochondria, production of ROS, decreased ATP content and cell viability, and reduced number of phagocytic mitochondria are associated with diabetic nephropathy, which lead to the destruction of glomeruli, renal tubules, blood vessels, and interstitium (Czajka et al., 2015; Palikaras et al., 2015; Wei et al., 2019). This suggests that dysregulation of mitophagy may be involved in the pathogenesis of diabetic nephropathy. Here, we review the detailed molecular mechanisms of mitophagy and highlight the role of mitophagy in the pathogenesis and treatment of DKD.

### Mitophagy

Autophagy (AP) is a conserved intracellular process in which damaged or redundant organelles are degraded by lysosomes (Tuttle et al., 2014). Mitophagy is the selective removal of mitochondria by autophagy to maintain mitochondrial content of cells and to ensure quality control (Galluzzi et al., 2017; Guan et al., 2018). Our understanding of mitophagy has come a long way since the coining of this term in 2005 (Lemasters, 2005). We now know that mitophagy is mediated by two different signaling pathways—the PINK1/Parkin pathway (Novak, 2012) and the mitophagy receptor pathway. The mitophagy receptor consists of three components, viz., 1) Outer mitochondrial membrane (OMM) proteins, such as Bcl-2/E1B-19K-interacting protein 3-like (BNIP3L/NIX) (Novak et al., 2010), BCL-2/adenovirus E1B 19-kDa interacting protein 3 (BNIP3) (Hanna et al., 2012), FUN14 domain containing 1 (FUNDC1) (Liu et al., 2012), FK506 binding protein 8 (FKBP8) (Bhujabal et al., 2017), and Bcl-2-like protein 13 (Bcl2-L-13) (Otsu et al., 2015), autophagy/beclin 1 regulator 1 (AMBRA1) (Strappazzon et al., 2015); 2) mitochondrial membrane lipids such as cardiolipin (Chu et al., 2013); and 3) mitophagy receptors, such as prohibitin 2 (PHB2), which form an inner mitochondrial membrane (IMM) component (Wei et al., 2017).

### PINK1/Parkin Pathway

PTEN-induced kinase 1(PINK1) is a serine/threonine kinase (Nguyen et al., 2016), a member of the RBR E3 ligase family, which is usually located in the cytoplasm. Activation of the PINK1/Parkin pathway is currently considered to be the first and most studied regulatory mechanism in mitophagy (Sekine and Youle, 2018).

### PINK1 Degradation

**FIGURE 1:**
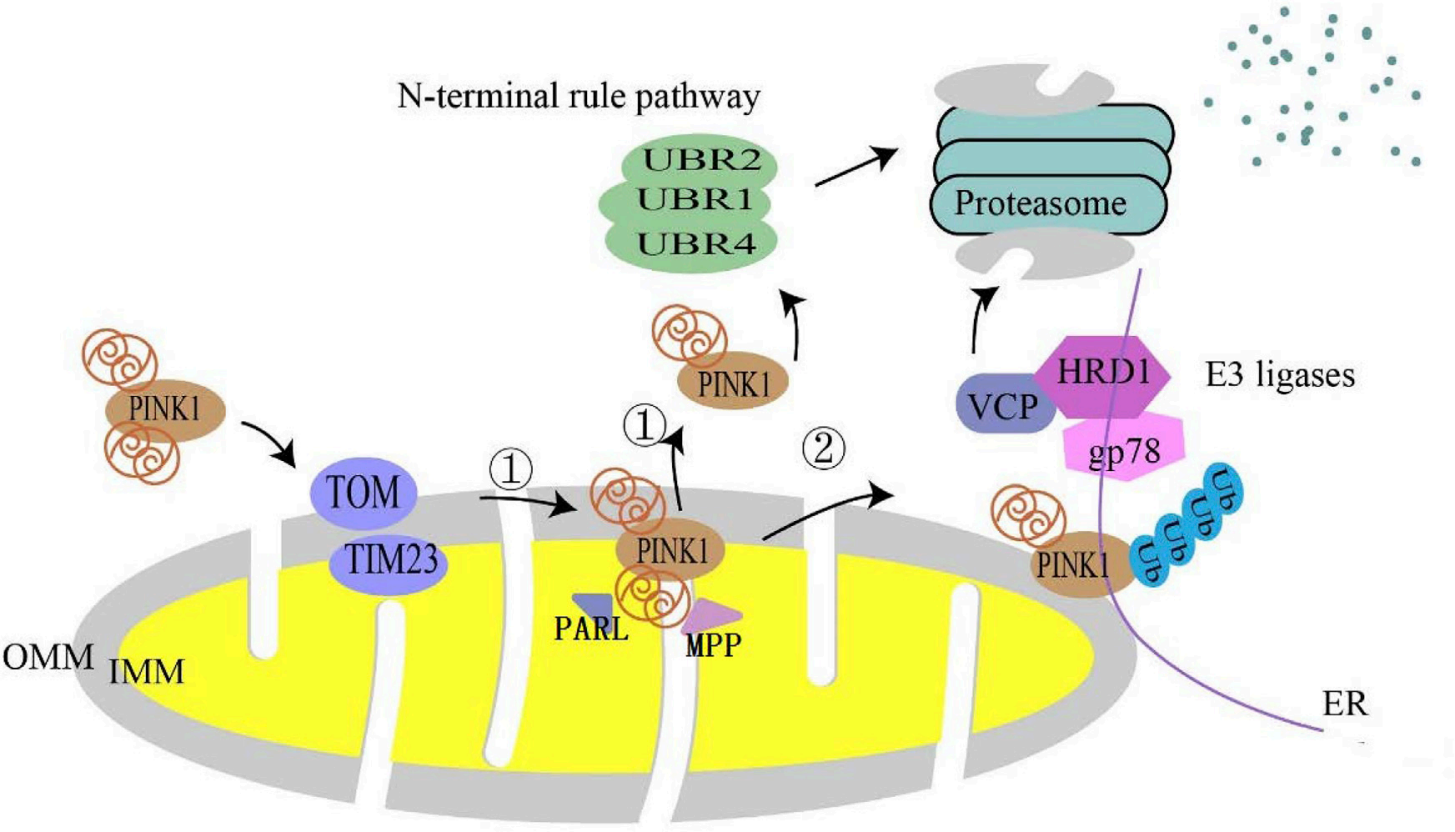
Degradation pathway of PINK1 in normal mitochondria. In normal mitochondria, PINK1 is imported into mitochondria utilizing the translocase complex of the outer membrane (TOM) and the translocase (TIM) of the inner membrane. In the inner mitochondrial membrane (IMM), ① is followed by proteolytic cleavage of full-length PINK1 by mitochondrial processing peptidase (MPP) processing and the intermembrane serine protease progerin-associated rhomboid protein (PARL), which cleaves full-length PINK1 into a 52 kDa fragment that is subsequently degraded by proteasomes *via* the conventional N-terminal pathway. ② It is also thought to be degraded at the mitochondrial–endoplasmic reticulum (ER) interface.

In healthy mitochondria, full-length PINK1 (64 kDa) is continuously directed to mitochondria based on the mitochondrial targeting sequence (MTS) in its N-terminus and is imported into the inner mitochondrial membrane by TOMM (translocase of the outer membrane) 40 and TIMM (translocase of the inner membrane) 23 complex (Sekine and Youle, 2018), and shortly after import PINK1 is cleaved by mitochondrial processing peptidase (MPP) and presenilins-associated rhomboid-like protein (PARL) (Spinazzi and De Strooper, 2016); MPP eliminates the MTS from the PINK1 structure and PARL cleaves PINK1 at the transmembrane structural domain (TMD) Ala103 to generate 52 kDa PINK1 (Deas et al., 2011; Yoo and Jung, 2018; Sekine, 2020). Cleaved PINK1 is transported to the cytoplasm and is degraded by proteasomes through the traditional N-terminal pathway (Yamano and Youle, 2013). In this process, E3 ubiquitin ligases, UBR1, UBR2, and UBR4, are responsible for the degradation of PINK1 (Yamano and Youle, 2013). It was recently reported that PINK1 could be degraded at the mitochondrial-endoplasmic reticulum (ER) interface through ubiquitin, endoplasmic reticulum, and proteasomal degradation (Guardia-Laguarta et al., 2019). The first two steps involved the endoplasmic reticulum (ER)-associated degradation (ERAD) pathway, which involved the E3 ligases gp78 and HRD1, and Valosin-containing protein (VCP) (Figure 1).

### Parkin Activation and Mitophagy

Parkin is inherently inactive and needs to be activated by PINK1. Autophosphorylation of PINK1 is a key event for the mitochondrial translocation of Parkin and its subsequent phosphorylation and activation (Zhuang et al., 2016). Misfolding or aggregation of mitochondrial proteins and excessive ROS levels in the mitochondrial matrix stimulate mitochondrial damage or depolarization. Thus, mitochondria fail to import PINK1 into IMM, which induces the accumulation and activation of PINK1 in the OMM (Jin et al., 2010). A recent study showed that ΔΨm deletion-dependent arrest of PINK1 import not only originated from Tim23 inactivation but was also closely associated with competition between TOM7, and OMA1 (Sekine et al., 2019). Phosphorylated PINK1 activates (Rasool et al., 2018) and phosphorylates ubiquitin on serine 65 (Ser65), resulting in the recruitment of Parkin from the cytoplasm to the damaged OMM and its partial activation (Matsuda et al., 2010; Vives-Bauza et al., 2010). Parkin recognizes phospho-Ser 65-ubiquitin (PSER65-UB) and translocates it to OMM proteins or the ubiquitin substrate, which provides more phosphorylated substrates for PINK1, and further recruits more Parkin, creating a positive feedback loop (Matsuda, 2016). PSER65-UB promotes the phosphorylation of UBLS65 residues in Parkin, which fully activates Parkin, ubiquitin, and many OMM proteins. This ultimately leads to the ubiquitination of specific proteins in the OMM and results in the recruitment of selective autophagy-adaptor proteins (Lazarou et al., 2015), such as nuclear dot protein 52 kDa (NDP52), optineurin (OPTN), P62/SQSTM1, neighbor of BRCA1 gene 1 (NBR1), and Tax1-binding protein 1 (TAX1BP1). TAX1BP1 served as a springboard for ring finger protein34 (RNF34) and mitochondrial antiviral signaling protein (MAVS) that facilitated RNF34-mediated autophagic degradation of MAVS through K27-linked ubiquitination (Xu et al., 2021). These proteins interact with microtubule-associated protein light chain 3 (LC3) to trigger the mitochondrial degradation process (Heo et al., 2015). Studies have shown that nipsnap homolog 1 (NIPSNAP1) and NIPSNAP2 are primarily mitochondrial matrix proteins that effectively act as “eat-me” signals for Parkin -dependent mitophagy, and NIPSNAP1 and/or NIPSNAP2 accumulate on the mitochondrial surface after mitochondrial depolarization, recruiting autophagy receptors as well as human Atg8 (autophagy-related 8)-family proteins to promote mitophagy (Abudu et al., 2019; Princely Abudu et al., 2019).

### Ubiquitin Substrate

The substrates for polyubiquitination by the active Parkin include the mitochondrial matrix protein methionine sulfoxide reductase B2 (MsrB2), mitochondrial Rho GTPase (Miro1) (Safiulina et al., 2019), F-box and WD repeat domain-containing 7 (Fbw7) (Yoon et al., 2017), phosphoglycerate dehydrogenase (PHGDH) (Liu et al., 2020a), mitofusin (MFN) 1 and 2 (Gegg et al., 2010), and voltage-dependent anion-selective channel protein 1 (VDAC1) (Geisler et al., 2010). The ubiquitinated substrates are degraded by proteasomes during the induction of mitophagy.

Specifically, MsrB2 is a necessary Parkin substrate. When mitochondria are severely damaged or ruptured, MsrB2 is released. It reduces Parkin methionine oxidation of MetO, which activates oxidized Parkin, and leads to the ubiquitination of MsrB2 by Parkin (Lee et al., 2019a). Additionally, some polyubiquitin substrates have multiple functions. Miro1 is considered a Ca ^2+^ biosensor. Recently, it was found that Miro is not only located in the mitochondria but also in peroxisomes. Besides acting as a substrate for parkin-dependent degradation, Miro can also initiate the recruitment of Parkin (Safiulina et al., 2019). Mitotic fusion proteins (MFN 1 and 2) are not only involved in the regulation of mitochondrial fission and fusion, but also in mitophagy (Gegg et al., 2010). Recently, it was reported that the PINK1/Parkin pathway is involved in the regulation of mitophagy and apoptosis through the induction of two different types of ubiquitination of VDAC1, and that VDAC1 polyubiquitination was involved in the regulation of Parkin-mediated mitophagy (Ham et al., 2020).

**FIGURE 2:**
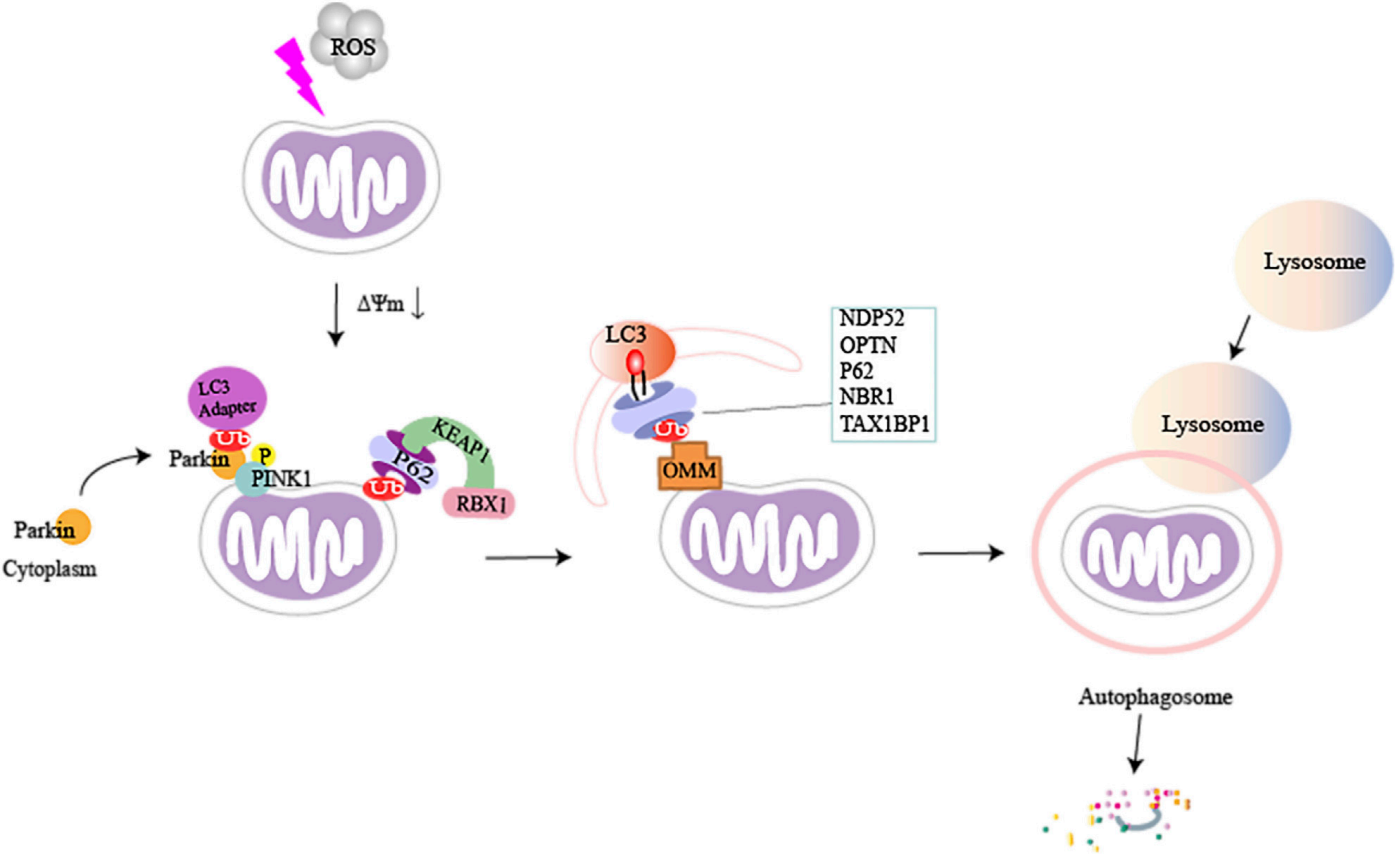
A brief illustration of PTEN-induced kinase 1 (PINK1) mediated mitophagy in damaged mitochondria. When cells are stimulated, the inner mitochondrial membrane undergoes sustained depolarization, which leads to a decrease in the mitochondrial transmembrane potential ΔΨm. PINK1 accumulates on the outer mitochondrial membrane and phosphorylates ubiquitin and Parkin, which causes Parkin to be recruited from the cytoplasm to the mitochondria. Activated Parkin can phosphorylate and ubiquitinate mitochondrial proteins (e.g., MFN1/2, MsrB2, Miro1, and Fbw7), and autophagic adaptor proteins can directly recognize polyubiquitin chains (p62, NDP52, OPTN, TAX1BP1, and NBR1) on damaged mitochondria by binding to microtubule-associated protein light chain 3 (LC3). This can recruit ubiquitinated substances into autophagosomes, subsequently forming mitochondrial autophagosomes, fusion with lysosomes, and ultimately break down of damaged mitochondria.

SQSTM1/P62 can also ubiquitinate mitochondrial proteins directly through the P62-KEAP1-RBX1 complex, which is independent of PINK1 and PRKN (Yamada et al., 2019). Similarly, it has been found that PINK1 can directly recruit OPTN/NDP52 through the ubiquitin-binding domain, independent of Parkin, and then it recruits UNC-51-like autophagy-activated kinase 1 (ULK1) in mitochondria to initiate mitophagy (Lazarou et al., 2015). In the absence of LC3, NDP52 associates with the ULK1 complex *via* focal adhesion kinase family-interacting protein of 200 kDa (FIP200), facilitated by TANK-binding kinase 1 (TBK1), and subsequently recruits the ULK1 complex to ubiquitinated cargo to initiate mitophagy (Vargas et al., 2019). This suggests that deficiency of the PINK1-dependent mitophagy factor can be adequately compensated through other means (Figure 2).

### Mitophagy Receptor Pathway

#### BNIP3 and NIX

BNIP3 and its homologous protein, Nix/BNIP3L, are members of the “BH3-only” Bcl-2 subfamily (Ney, 2015). Both BNIP3 and NIX contain LC3-interacting region (LIR) motifs that can bind to LC3, inducing mitophagy (Schwarten et al., 2009; Hanna et al., 2012).

Previous studies have shown that BINP3 is involved in autophagic cell death in apoptosis-competent cells under hypoxia (Azad et al., 2008). Phosphorylation of S17 and S24 in BNIP3 regulates the interaction between BNIP3 and LC3 during mitochondrial depolarization (Zhu et al., 2013), suggesting that kinases or phosphatases may play a role in modulating mitophagy.

NIX-dependent mitochondrial removal was first identified in mature erythrocytes (Schweers et al., 2007), and previous research demonstrated that phosphorylation of Ser34 and Ser35 in Nix enhances its interaction with LC3 and increases the recruitment of autophagic machinery to mitochondria (Rogov et al., 2017). Recent evidence suggests that LIR phosphorylation and NIX dimerization are novel molecular mechanisms for the activation of NIX, wherein Ser212, the major amino acid residue in the C-terminus, is responsible for the dimerization of NIX (Marinkovic et al., 2020). These structural studies provide important information for exploring drugs that target NIX.

#### FUNDC1

FUNDC1 is a new OMM protein that promotes mitochondrial fragmentation and mitophagy during hypoxia (Liu et al., 2012; Zhang et al., 2016a; Zhang et al., 2017). Recently, it was shown that FUNDC1 could recruit dynamin 1-like (DNM1L) to drive mitochondrial fission (Wu et al., 2016). Dephosphorylation of Ser13 of FUNDC1 enhances its interaction with DNM1L, thus enhancing mitophagy (Chen et al., 2016). In addition, in a model of ischemic acute kidney injury, the protective effect of Fundc1-dependent mitophagy could be exerted via the suppression of Drp1-mediated mitochondrial fission (Wang et al., 2020a). FUNDC1-mediated mitophagy is greatly affected by the phosphorylation status of FUNDC1 (Lv et al., 2017). Under normal conditions, CK2 kinase-induced Ser13 phosphorylation of the LIR motif and SRC kinase-induced Tyr18 phosphorylation of the LIR motif inhibit FUNDC1-mediated mitophagy, whereas Ser17 phosphorylation promotes mitophagy (Kuang et al., 2016). Under loss of mitochondrial membrane potential or hypoxia, FUNDC1 undergoes dephosphorylation due to loss of SRC and CK2 kinase activity and activation of phosphatases including phosphoglycerate mutase 5 (PGAM5) and ULK1 (Wu et al., 2014a; Chen et al., 2014). Dephosphorylated FUNDC1 interacts with LC3 through its typical LC3 binding motif Y 18) XXL 21) to induce mitophagy. FUNDC1 was shown to be a substrate for mitochondrial ubiquitin ligase (MARCH5), an E3 ligase localized to the OMM. In addition, the MARCH5-FUNDC1 axis is capable of fine-tuning hypoxia-induced mitophagy by a mechanism that may be related to the regulation of FUNDC1 ubiquitination and degradation (Chen et al., 2017a). B-cell lymphoma-2-like 1 (BCL2L1) inhibits PGAM5 activity (Wu et al., 2014b), whereas syntaxin 17 (Stx17) promotes interaction between PGAM5 and FUNDC1. FUNDC1 can also bind to inositol 4-inositol-5-trisphosphate type 2 receptor (IP3R2) in the endoplasmic reticulum, activating calcium-dependent cAMP responsive element binding protein (CREB) to regulate fission protein 1 (Fis1) at the transcriptional level and promote mitophagy (Wu et al., 2017).

### Regulatory Factors and Interaction of BINP3 and FUNDC1 Expression

The process of mitophagy induced by BINP3, FUNDC1, and Parkin may not occur independently. These factors not only promote their own recruitment through positive feedback loops but also promote the recruitment of other pro-mitophagy factors. BNIP3 and NIX can promote the recruitment of Parkin to mitochondria (Ding et al., 2010; Lee et al., 2011). BNIP3 can suppress the proteolysis of PINK1 and facilitates the accumulation of full-length PINK1 on the OMM (Zhang et al., 2016b). NIX can be used as an alternative mediator of mitophagy (Koentjoro et al., 2017) restoring mitochondrial function under PINK1 or Parkin deficiency.

As of date, many factors have been identified as BNIP3 transcription factors; these include hypoxia-inducible factor-1 subunit α (HIF-1 α), MIR-145 (Liu et al., 2019), p53 (Feng et al., 2011), and Brain and muscle ARNT-like protein 1 (BMAL1) (Li et al., 2020). Specifically, insulin-like growth factor I (IGF-1) signal transduction promotes tumorigenesis. IGF-1-induced BNIP3-dependent mitophagy is a stress-protective response in cancer cell lines and mouse embryonic fibroblasts (MEFs). This process also requires the nuclear factor erythroid-2 related factor 2 (Nrf2) to play a critical through HIF-1 α and nuclear factor erythroid 2-like 1 (NRF1) (Riis et al., 2020). The BDNF/TrkB/HIF-1 α/BNIP3 signal transduction pathway can regulate the mitophagy in bone marrow endothelial cells (Jin et al., 2019), which complements the upstream signaling of HIF-1 α/BNIP3. In cardiomyocytes, the circadian gene, *BMAL1,* was observed to bind to the E-box element in the BNIP3 promoter, thereby directly regulating BINP3 transcription (Li et al., 2020).

Macrophage stimulating factor 1 (Mst1), a new type of mitophagy upstream regulator, is involved in the regulation of multiple mitophagy pathways. This was verified through a series of tests. In colorectal cancer, overexpression of Mst1 can activate the JNK/p53 pathway, increase p53 phosphorylation, and eventually inhibit BNIP3-related mitophagy (Li et al., 2018a). In fatty liver disease, it was found that Mst1 gene knockout reverses Parkin-related mitotic phagocytosis, which regulates Parkin expression through the AMP-activated protein kinase (AMPK) pathway (Zhou et al., 2019a). p53 is a mitotic regulator that can inhibit the transcription and expression of BNIP3 and FUNDC1. It was shown that BNIP3-mediated mitophagy is regulated by the NR4A1/DNA-PKcs/p53 axis in non-alcoholic fatty liver disease, resulting in mitophagy arrest (Zhu et al., 2020). Further experiments showed that the Sirt3/ERK/CREB signal transduction axis is also involved in the regulation of BNIP3-mediated mitophagy. Thus, it inhibits mitochondrial-dependent apoptosis of hepatocytes (Li et al., 2018b). Surprisingly, it was found that the NR4A1/DNA-PKcs/p53 transduction axis also activates Drp1-related mitochondrial fission and limits FUNDC1-related mitophagy in alcohol-related liver disease (ARLD). This axis is also related to the increase in the expression of CK2 by p53 (Zhou et al., 2019b).

In mammals, the mitogen-activated protein kinase (MAPK) pathway consists of three parts—the extracellular signal-regulated kinase (ERK), c-Jun N-terminal kinase (JNK), and p38 kinase pathway. Among these, there is evidence that the ERK and JNK pathways are involved in the regulation of FUNDC1-dependent mitophagy. In myocardial ischemia-reperfusion injury, Mst1 inactivates FUNDC1-related mitophagy by downregulating the MAPK/ERK/CREB pathway (Yu et al., 2019). In laryngeal cancer cells, upregulation of FUNDC1 expression can be achieved by activating Erk1/2 signaling (Hui et al., 2019), and JNK and ERK MAPK activation can also trigger BNIP3-induced mitophagy. This was also confirmed by UVB radiation-mediated accumulation of ROS in human primary epidermal keratinocytes (HPEK) (Moriyama et al., 2017). In TNF-α–induced mouse microglial BV-2 cells, the MAPK/ERK/Yap signal pathway was involved in MA-5-mediated upregulation of BNIP3 expression (Lei et al., 2018).

Sirtuin 3 (SIRT3) is a mitochondrial deacetylase in neonatal mouse cardiomyocytes treated with high amounts of glucose. Overexpression of SIRT3 significantly increased the deacetylation of Forkhead box class O 3a (FoxO3a) as well as the expression of Parkin and autophagy marker (LC3B) (Yu et al., 2017). The study found that when SIRT3 was blocked, the deacetylation of p53 was not maintained, increasing p53–Parkin binding and blocking Parkin depolarized mitochondrial translocation (Li et al., 2018c). In addition, Newcastle disease virus infection induces SIRT3 loss *via* PINK1-PRKN-dependent mitophagy to reprogram energy metabolism (Gong et al., 2021). In CATH.a cells, mitochonic acid 5 (MA-5) increased the expression of SIRT3 through the AMPK pathway and promoted Parkin-related mitophagy (Huang et al., 2019).

**FIGURE 3:**
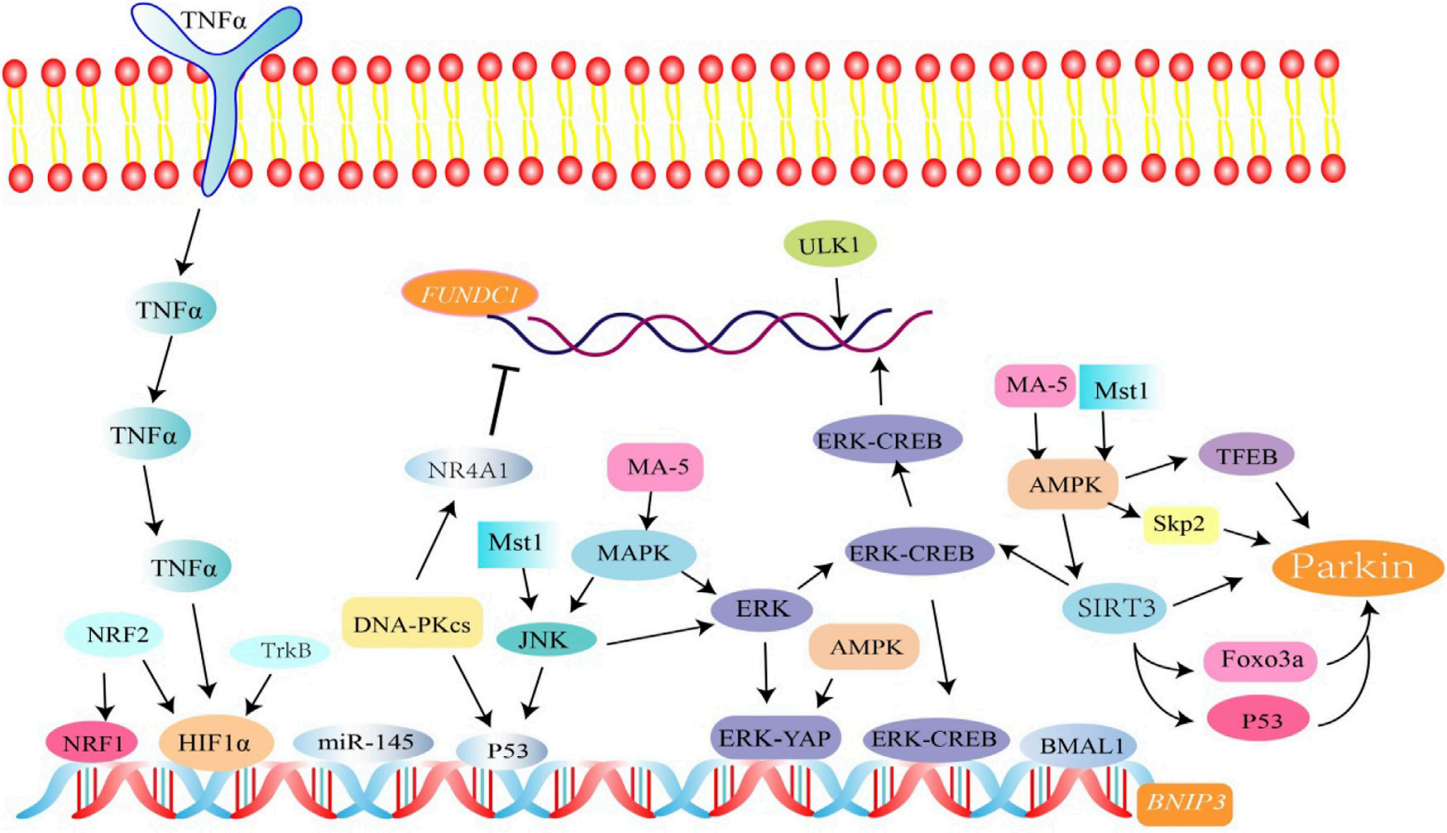
Related factors regulate FUNDC1, BINP3, and Parkin-mediated mitophagy. Among them, SIRT3 activates Parkin-associated mitophagy by mediating FOXO3a and p53 deacetylation. SIRT3 is involved in the regulation of BNIP3-mediated mitophagy through the ERK-CREB signal transduction axis. p53 is involved in the regulation of mitophagy. The NR4A1/DNA-PKcs/p53 pathway inhibits FUNDC1 and BINP3-mediated mitophagy.

AMPK, a heterotrimeric complex, is an important cellular energy receptor and metabolic switch. There is evidence that the AMPK signaling pathway promotes nuclear translocation of the transcription factor EB (TFEB) and induces Parkin-related mitophagy (Cao et al., 2020). In addition, AMPK can also promote phosphorylation of Parkin by activating S-phase kinase-associated protein 2 (Skp2) (He and Gu, 2018). In cardiomyocytes, overexpression of the AMPKα 2 subtype can phosphorylate Ser495 PINK1 to eliminate damaged mitochondria (Wang et al., 2018a). AMPK mediates phosphorylation of TBK1 through the ULK1 complex, which enhances the binding ability of autophagy receptors (such as OPTN) to the ubiquitin cargo (Seabright et al., 2020) (Figure 3).

### FKBP8

FKBP8 belongs to the FK506 binding protein family and is a new type of mitochondrial receptor. It recruits LC3A to damaged mitochondria in an LIR-dependent manner to promote mitophagy, which is unrelated to the PINK1-Parkin-induced mitophagy pathway (Bhujabal et al., 2017).

### Bcl2-L-13

Bcl2-L-13 is an OMM protein. When expressed in yeast, it can be used as a mitochondrial receptor to compensate for the function of mitophagy receptor, Atg32 (Mao et al., 2011). After mitochondrial depolarization, Bcl2-L-13 recruits the ULK1 complex, which is composed of ULK1, ATG13, FIP200, and ATG101. Thereafter, LC3B is recruited to the OMM. ULK1 induces mitophagy through interaction between the LIR motif in the Bcl2-L-13-ULK1 complex and LC3B (Murakawa et al., 2015; Murakawa et al., 2019).

### AMBRA1

In an ischemia/reoxygenation model, AMBRA1, the E3 ubiquitin ligase, HUWE1, and IKKα kinase together regulate mitophagy. AMBRA1 acts as a cofactor for HUWE1 activity, promotes interaction between HUWE1 and MFN2, leading to the ubiquitination and degradation of MFN2, which ultimately affects mitophagy. Moreover, IKKα can phosphorylate AMBRA1 on Ser1014, allowing the AMBRA1-LIR pattern to interact with LC3 (Di Rita et al., 2018). AMBRA1 is also thought to enhance the PINK1/PARK2-dependent mitophagy (Strappazzon et al., 2015). Myeloid cell leukemia-1 (MCL1), a member of the BCL2 family, is a negative regulator of AMBRA1-dependent mitophagy, which inhibits the recruitment of HUWE1 to the mitochondria. When MCL1 is phosphorylated by GSK-3β at a conserved GSK-3 phosphorylation site (S159), the stability of MCL1 is disrupted, which initiates AMBRA1-induced mitophagy and ultimately leads to the degradation of MCL1 (Strappazzon et al., 2020).

### PHB2

PHB2, a highly conserved membrane scaffolding protein, was identified as a novel inner membrane mitophagy receptor that mediates mitophagy (Wei et al., 2017). Recent evidence suggests that PHB2 promotes PINK1-PRKN/Parkin-dependent mitophagy through the PARL-PGAM5-PINK1 axis. During mitochondrial depolarization or damage, PHB2 interacts with PARL to protect the structural integrity of PGAM5, thereby, retaining PINK1 on the OMM, which subsequently recruits Parkin to the damaged mitochondria, initiating mitophagy (Lahiri and Klionsky, 2017; Yan et al., 2020). Bax inhibitor 1 (BI1) could stabilize PHB2 and promote its retention in mitochondria, thus, preserving mitochondrial homeostasis (Wang et al., 2020b).

### Cardiolipin

**FIGURE 4:**
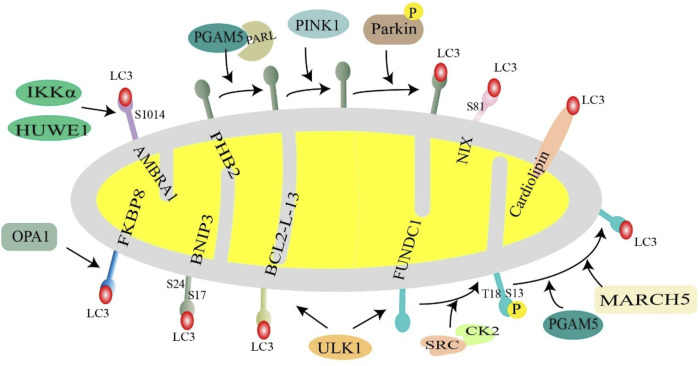
Receptor-mediated mitochondrial phagocytosis. Mitochondrial membrane proteins (NIX, BNIP3, FUNDC1, AMBRA1, BCL2L13, PHB2, and FKBP8) and lipids (cardiolipin) interact with LC3 and then recruit autophagosomes to mitochondria to initiate mitophagy. Under hypoxic conditions, BNIP3 is phosphorylated at S17 and S24, and NIX is phosphorylated at Ser81, favoring interaction with LC3. Normally, FUNDC1 is phosphorylated at S13 and T18 by CK2 kinase and SRC kinase, respectively, to inhibit FUNDC1-mediated mitophagy, when mitochondria are stimulated, UNC-51-like autophagy-activated kinase 1 (ULK1) and E3 ubiquitin ligase March5, PGAM5 phosphatase dephosphorylate FUNDC1. BCL2L13 recruits ULK1 to form a Bcl2-L-13-ULK1 complex that binds to LC3 through its LIR motif. PHB2 in the inner mitochondrial membrane acts as a mitochondrial receptor and cooperates with PARL, PGAM5, PINK1, and Parkin to regulate mitophagy. AMBRA1 is phosphorylated at S1014 in response to two key factors, HUWE1 and IKKα, enhancing the interaction with LC3. FKBP8 binds to LC3 in an LIR-dependent manner. Cardiolipin translocates to the outer mitochondrial membrane and directly interacts with LC3 to promote mitophagy.

Cardiolipin is a diphosphatidylglyceride that is distributed in the IMM. Rotenone, 6-hydroxydopamine, and other pre-mitochondrial stimuli cause cardiolipin to be externalized to the mitochondrial surface. Cardiolipin directly interacts with microtubule-associated protein 1 light chain 3 (MAP1LC3) to induce mitophagy for clearing damaged mitochondria (Chu et al., 2013). This suggests that cardiolipin can act as an important “I eat” signal to regulate mitochondrial cell death and survival (Figure 4).

### Mitophagy and Renal Cells in DKD

**FIGURE 5:**
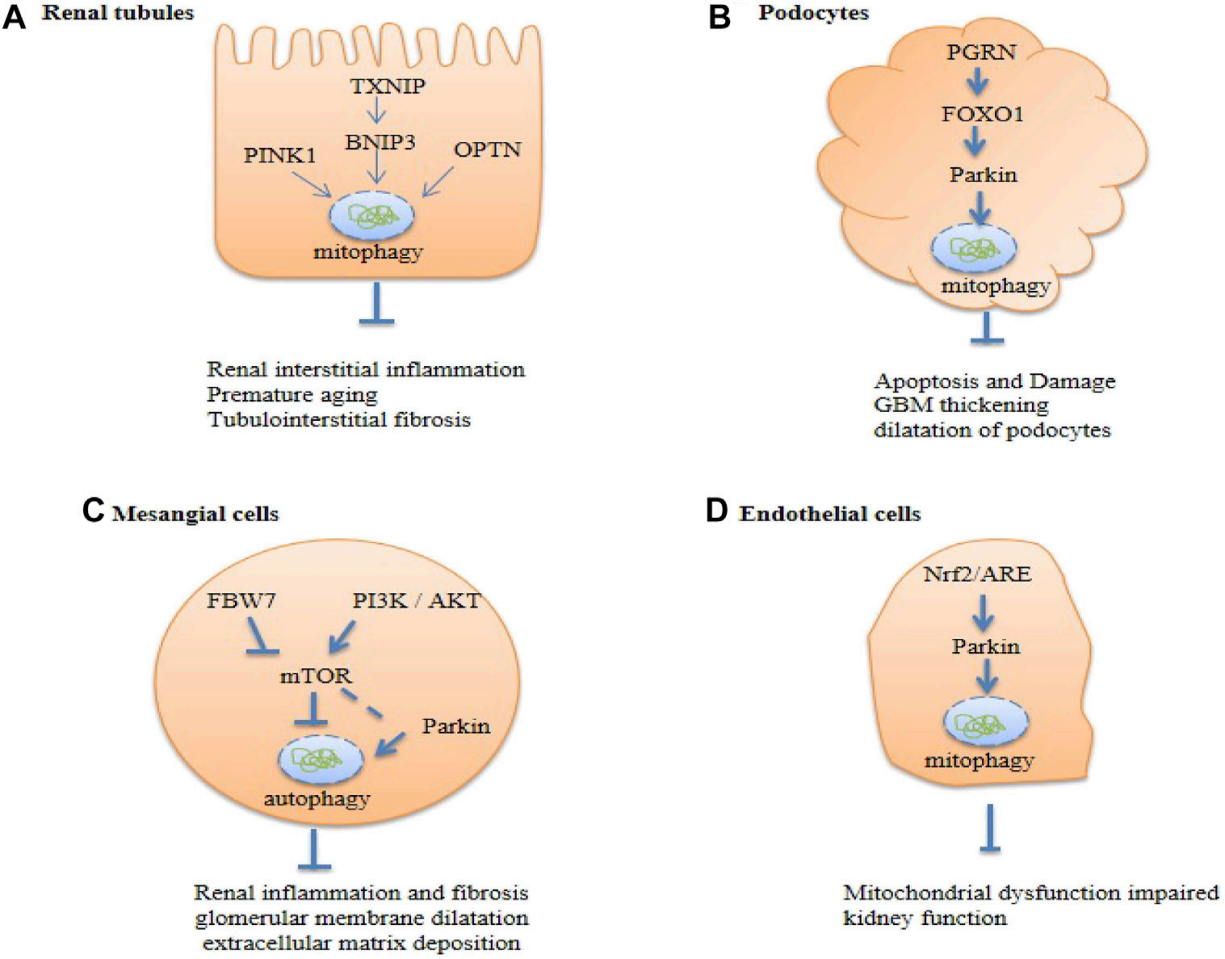
Summary of renal cell autophagy-mediated pathways in diabetic kidney disease (DKD). Txnip, Thioredoxin-interacting protein; OPTN, Optineurin; PGRN, Progranulin; FBW7, F-box and WD repeat domain-containing protein 7; PI3K, The phosphoinositide 3- kinase; Akt, protein kinase B; Nrf-2, nuclear factor erythroid 2-related factor 2; mTOR, the mammalian target of rapamycin.

The mechanisms of mitophagy in kidney function and pathology remain largely understudied. We are only beginning to appreciate the complex cellular process of mitophagy. A growing body of evidence implicates the importance of mitophagy in the maintenance of kidney homeostasis and pathogenesis. Much of the current insight has been gained from investigations on renal cells in culture and from complementary studies on animal models. The regulation and function of mitophagy in the kidney are likely cell type- and context-specific. Below, we discuss studies on four resident renal cell types, viz., tubular epithelial cells, podocytes, glomerular mesangial cells, and endothelial cells (Figure 5). These highly specialized cell types are targets for diabetic kidney injury.

### Renal Tubules and Mitophagy in DKD

In renal tubular epithelial cells (RTECs) from mice treated with high glucose (HG) and in renal biopsies from DKD patients, diminished levels of mitophagy were observed (Chen et al., 2018). Additionally, there is evidence for diminished mitophagy in the renal tissue of diabetic mice, with decreased expression of mitochondrial PINK1, Parkin, LC3II (mito-LC3II), Beclin1, and Atg5 (Feng et al., 2018). Findings in primary human renal epithelial cells *in vitro* demonstrate that mitochondrial quality control can be perturbed by mitophagy mediated through PINK/Parkin (Chen et al., 2018; Chen et al., 2020; Kang et al., 2020; Zhao and Sun, 2020). In diabetic nephropathy models, Parkin knockout leads to many injury features, such as apoptosis, inflammation, fibrosis, premature senescence of RTECs, and decreased renal function (Chen et al., 2020). The mitochondrial division/mitophagy inhibitor, Mdivi-1, could accelerate senescence in RTECs (Chen et al., 2018), whereas in HG-stimulated HK2 and LLC-PK1 cells, overexpression of MIOX, a tubule-specific enzyme, inhibits PINK1/Parkin-induced mitophagy, which leads to increased renal ROS generation and tubulointerstitial injury in diabetic mice (Zhan et al., 2015). *In vitro* studies showed dysregulation of mitophagy in HG-induced proximal tubular cells, and TXNIP siRNA restored tubular mitophagy by inhibiting the mTOR signaling pathway and expression of BNIP3 (Huang et al., 2016). A large body of evidence indicates that impaired mitophagy may play a fundamental role in the pathogenesis of DKD.

Interestingly, overexpression of PINK1 in mouse RTECs failed to attenuate mitochondrial dysfunction and cellular senescence. In contrast, increased mitochondrial formation, decreased the accumulation of mitochondrial ROS (MtROS), and activation of mitophagy was observed with overexpression of OPTN, which ultimately alleviated premature tubular cell aging (Chen et al., 2018). In addition, decreased OPTN expression leads to the development of tubulointerstitial inflammation and the mechanism may be related to the accumulation of damaged mitochondria and activation of the NLRP3 inflammasome, which in turn leads to the cleavage of caspase-1 and IL-1β and a significant increase in the release of IL-1β and IL-18 (Chen et al., 2019). This suggests that OPTN may be the most important protein regulator of mitophagy in RTECs.

### Podocytes and Mitophagy in DKD

The mitochondrial LC3II/LC3I ratio and levels of PINK1, Parkin, and Mfn1 proteins were downregulated, whereas p62 protein levels were upregulated in renal tubular epithelial cells of diabetic mice, podocytes of diabetic mice, and high glucose-treated MPC5 cells (Zhan et al., 2015; Sun et al., 2019; Guo et al., 2020; Han et al., 2021). Additionally, glomerular basement membrane thickening and dilatation of podocytes were observed in the kidneys of diabetic mice, suggesting that mitophagy of podocytes is inhibited and affects the progression of diabetic nephropathy (Li et al., 2017a; Guo et al., 2020). Silencing of Parkin inhibited mitophagy and significantly increased apoptosis of podocytes and production of MtROS, induced with palmitic acid (Jiang et al., 2020).

A recent report provided evidence that mitochondrial phagocytosis may be inhibited by the downregulation of the secreted glycoprotein, PGRN (Zhou et al., 2019c). The expression of PGRN was significantly decreased in the kidneys of both DKD patients and diabetic mice. Reduced PGRN expression leads to the inhibition of the transcription factor, FoxO1, dependent on STAT1, resulting in the inhibition of the expression of the mitophagy-associated proteins, PINK1 and Parkin, which leads to mitochondrial dysfunction in the kidney under HG conditions (Zhou et al., 2019d). In addition, FoxO1 can regulate the PINK1/Parkin-dependent mitophagy activity. Overexpression of FoxO1 could ameliorate apoptosis and injury in a cultured immortalized mouse podocyte cell line (CIMP) and in mouse glomerular mesopodia induced by streptozotocin (STZ) (Zhou et al., 2019d). Knockdown of lncRNA SNHG17 promotes the formation of autophagic vacuoles and Parkin-mediated mitophagy by regulating the degradation of Mst1 (Guo et al., 2020).

These studies have shown that in diabetic kidneys, factors, such as PGRN, FoxO1, and lncRNA SNHG17, are involved in PINK1/Parkin-induced mitophagy through different pathways. These weaken the protective function of mitophagy, which leads to diabetic kidney disease.

### Mesangial Cell and Mitophagy in DKD

Increasing evidence shows that mitophagy plays an important role in the pathogenesis of DKD. The expression of autophagy markers, LC3I/LC3I and p62, was observed in rat mesangial cells in a time-dependent manner (Chen et al., 2017b). It was observed that Parkin was recruited to the damaged mitochondria, and the production of autophagic vacuoles was significantly increased (Xu et al., 2016). Similarly, decreased mRNA expression of autophagy-associated proteins, Beclin1, PINK1, and Parkin, was observed in renal mesangial cells (RMC) under HG conditions. Stimulation of mTOR/PINK1/Parkin-mediated mitophagy can improve renal inflammation, glomerular membrane dilatation, and extracellular matrix deposition (Wen et al., 2020). Stimulation of BNIP3/Nix signal transduction also has the same intervention effect (Jin et al., 2020).

Several studies have shown that the activation of the PI3K/AKT/mTOR pathway is involved in the autophagy of glomerular mesangial cells, which exhibited increased phosphorylation of mTOR (Lee et al., 2019b), PI3K, Akt, and mTOR STZ-induced type 1 diabetic nephropathy (T1DN) (Wang et al., 2020c). In another study, FBW7 was shown to be an upstream stimulator of mTOR and overexpression of FBW7 could inhibit mTOR signaling to increase autophagy and alleviate DKD (Gao et al., 2019). Triptolide (TP) can restore autophagy and reduce fibrosis through the miR-141-3p/PTEN/Akt/mTOR pathway in DKD rats and human mesangial cells cultured with under HG conditions (Li et al., 2017b). Ursolic acid can inhibit the activation of mTOR to reduce the accumulation of extracellular matrix and improve hypertrophy and proliferation of cells (Lu et al., 2015). These studies suggest that autophagy and mitophagy play roles in renal protection.

### Endothelial Cells and Mitophagy in DKD

As of date, only a few studies have been conducted on the role of mitophagy in endothelial cells. In db/db mice, an animal model of type 2 diabetes, glomerular LC3 accumulation is reduced and is accompanied by decreased accumulation of ATP and mtROS. When exposed to HG, mitochondrial glomerular endothelial cells (mGECs) were found to have decreased levels of LC3 - II, PINK, and parkin (Sun et al., 2019). Glomerular apoptosis was significantly ameliorated in db/db mice using the mitophagy-activating factor Torin1, suggesting that mitophagy plays an important and possibly therapeutic role in this disease. Activation of Nrf2/ARE signaling promotes mitophagy in glomerular endothelial cells may restore diabetic nephropathy-induced mitochondrial dysfunction and impaired renal function (Sun et al., 2019). In diabetic conditions, the mechanism underlying weakened mitophagy leading to glomerular lesions needs to be investigated further.

### Prospects of Targeting Mitophagy Therapy in DKD

Given the key role of mitophagy in DKD, specific interventions targeting mitophagy to protect and restore mitochondrial function have become promising strategies for preventing, treating, and mitigating DKD. As of date, various compounds and Chinese herbal decoctions targeting mitophagy have been shown to protect the kidney from damage in DKD.

A recent study showed that miR-379 deletion ameliorates features of DKD by enhancing adaptive mitophagy *via* FIS1 (Kato et al., 2021). In additon, MitoQ and CoQ10 are mitochondria-targeting antioxidants that enhance mitophagy and clearance of damaged mitochondria in the kidney. CoQ10 modulates mtROS and Nrf2/ARE pathways to increase glomerular mitophagy and improves renal function and mitochondrial homeostasis in db/db mice (Sun et al., 2019). MitoQ consists of ubiquinone (oxidized CoQ10) molecules conjugated to triphenylphosphonium (TPP) cation moiety, which can effectively prevent mitochondrial oxidative damage when compared with the prevention achieved by CoQ10 (Ross et al., 2005; James et al., 2007; Murphy and Smith, 2007). Renal function and tubular interstitial fibrosis in Ins2+/-AkitaJ mice in a type 1 diabetes model were improved by oral administration of MitoQ (Chacko et al., 2010), and in addition, there is evidence that MitoQ treatment also improved tubular injury and apoptosis in HG conditions, which is closely related to upregulation of Nrf2 and PINK/Parkin pathways to regulate mitochondrial phagocytosis in renal tubules (Xiao et al., 2017).

D-gluconate is a potential pharmacological inhibitor of MIOX. It has a renal protective effect in DKD. D-gluconate therapy reversed STZ-induced inhibition of mitophagy in diabetic mice and improved the integrity of renal tubule cells (Zhan et al., 2015). It was demonstrated that metformin, a classic antidiabetic agent, protects renal epithelial cells stimulated with HG *in vitro* by activating PP2A and inhibiting NF-κB to reverse the expression of Parkin and mitophagy (Zhao and Sun, 2020). These results suggest that activation of mitophagy, especially of the PINK1/Parkin pathway, is an attractive target for improving diabetes-induced renal damage.

In addition, traditional Chinese medicine (TCM) has been widely used in the treatment of diabetes and its complications and has achieved good results (Tian et al., 2019). However, there are relatively few studies on the potential therapeutic mechanisms of TCMs against diabetic nephropathy. Intriguingly, in db/db mice, AstragalosideIV (AS-IV) treatment reduced the expression of mitophagy-related proteins, PINK1, Parkin, p-Parkin (Ser65), and LC-3II, in the kidney, thereby, reducing proteinuria and promoting recovery from renal injur (Liu et al., 2017). Similarly, treatment with Huangqi-Danshen decoction (HDD) improved glomerular hypertrophy and increased glomerular mesangial cells in db/db mice (Liu et al., 2020b). This suggests that the mechanism underlying the effect of TCM against DKD may involve inhibition of the mitophagy activity of renal mitochondria.

Mitophagy inducers, such as resveratrol, rapamycin, quercetin, or berberine, which trigger multiple signaling pathways, are perceived as potential protective molecules against DKD. For example, resveratrol turns on the expression of Sirt, Beclin1, and BNIP3 (Ajoolabady et al., 2020). Rapamycin, a pharmacological inhibitor of mTOR, stimulated autophagy and mitophagy, and alleviated nephrotoxicity by restoring the expression of PINK1 and Parkin (Wang et al., 2018b). Quercetin can alleviate kidney fibrosis by reducing the senescence of renal tubular epithelial cells through the SIRT1/PINK1/mitophagy axis (Liu et al., 2020c). Berberine alleviates cisplatin-induced acute kidney injury by regulating mitophagy via the PINK1/Parkin pathway (Qi et al., 2020). To this end, the search for inducers of mitophagy, with minimal side effects, is pertinent.

Mitophagy is considered to be a protective mechanism under pathological conditions. However, with the progression of diabetic nephropathy, mitophagy becomes overburdened or impaired and mitochondrial fragments accumulate, leading to cell death. TCM treatment is counterintuitive to the regulation of mitophagy activity in DKD kidney by MITOQ, CoQ10, and D-gluconate. A possible explanation is that mitophagy plays a role in kidney protection, but its long-term activation may lead to cell damage. This hypothesis needs to be tested in clinical trials (Table 1).

**TABLE 1:** Summary of drugs aimed at improving mitochondrial dysfunction in DKD.

Agent	Mechanism	Model	Influence factor	Effect on DKD and repair	Effect on mitophagy	Pub. year	References
d-gluconic	inhibitor of MIOX	diabetic mice	PINK1, MIOX, ROS	Attenuated DKD	Promote	2015	Zhan et al. (2015)
CoQ10	Mitochondria-targeted antioxidant	db/db type 2 diabetic mice	MtROS, Nrf2/ARE	Attenuated DKD	Promote	2019	Sun et al. (2019)
MitoQ	Mitochondria-targeted antioxidant	db/db mice	Nrf2/Keap1 and PINK/Parkin	Attenuated DKD	Promote	2017	Xiao et al. (2017)
HDD		db/db mice	PINK1/Parkin	Reduces urinary albumin excretion and improves kidney injury	Restrain	2020	Liu et al. (2020b)
AS-IV		db/db mice		Attenuated DKD	Restrain	2017	Liu et al. (2017)
metformin			PP2A, NF-κB, Parkin	Attenuated DKD	Promote	2020	Zhao and Sun (2020)

## Conclusion

There is no definitive cure for diabetic nephropathy. The main treatment options for patients with diabetic nephropathy are to reduce cardiovascular risk, control blood glucose, blood pressure, blood lipids, and institute lifestyle changes. Drugs used to treat diabetes such as the renin-angiotensin system (RAS) inhibitors, including angiotensin-converting enzyme inhibitor (ACEI) and angiotensin receptor blocker (ARB) (Lv et al., 2012; Miyazaki et al., 2015). Drugs that control blood sugar include sodium-glucose cotransporter-2 inhibitors (SGLT2i) (Fioretto et al., 2016; Kawanami et al., 2017), glucagon-like peptide-1 receptor agonists (GLP-1 RAs), and dipeptidyl peptidase IV (DPP-4) inhibitors (Inzucchi et al., 2012; Pugliese et al., 2019); however, side effects of these drugs have been reported (Seger et al., 2021). Additionally, early prevention or slowing down the progression of DKD cannot effectively prevent the development of end-stage renal disease; therefore, it is particularly important to find new therapeutic targets. There is evidence that mitochondrial-targeted therapy for diabetic nephropathy may have a renal protective effect. However, related studies have shown that excessive mitophagy may also lead to cell damage. Future studies are needed to reveal the exact role of mitophagy in the renal tubules and glomeruli in DKD. We also need to explore the roles of other cellular processes associated with mitophagy in DKD, which will further promote our understanding of the molecular mechanisms behind DKD and the discovery of potential treatment methods.
